# Parasite-Derived MicroRNAs in Host Serum As Novel Biomarkers of Helminth Infection

**DOI:** 10.1371/journal.pntd.0002701

**Published:** 2014-02-20

**Authors:** Anna M. Hoy, Rachel J. Lundie, Alasdair Ivens, Juan F. Quintana, Norman Nausch, Thorsten Forster, Frances Jones, Narcis B. Kabatereine, David W. Dunne, Francisca Mutapi, Andrew S. MacDonald, Amy H. Buck

**Affiliations:** 1 Centre for Immunity, Infection and Evolution, Ashworth Laboratories, University of Edinburgh, Edinburgh, United Kingdom; 2 The Walter and Eliza Hall Institute of Medical Research, Parkville, Victoria, Australia; 3 Division of Pathway Medicine, The University of Edinburgh, Edinburgh, United Kingdom; 4 Department of Pathology, University of Cambridge, Cambridge, United Kingdom; 5 Vector Control Division, Ministry of Health, Kampala, Uganda; 6 Manchester Collaborative Centre for Inflammation Research, The University of Manchester, Manchester, United Kingdom; Centers for Disease Control and Prevention, Atlanta, United States of America

## Abstract

**Background:**

MicroRNAs (miRNAs) are a class of short non-coding RNA that play important roles in disease processes in animals and are present in a highly stable cell-free form in body fluids. Here, we examine the capacity of host and parasite miRNAs to serve as tissue or serum biomarkers of *Schistosoma mansoni* infection.

**Methods/Principal Findings:**

We used Exiqon miRNA microarrays to profile miRNA expression in the livers of mice infected with *S. mansoni* at 7 weeks post-infection. Thirty-three mouse miRNAs were differentially expressed in infected compared to naïve mice (>2 fold change, p<0.05) including miR-199a-3p, miR-199a-5p, miR-214 and miR-21, which have previously been associated with liver fibrosis in other settings. Five of the mouse miRNAs were also significantly elevated in serum by twelve weeks post-infection. Sequencing of small RNAs from serum confirmed the presence of these miRNAs and further revealed eleven parasite-derived miRNAs that were detectable by eight weeks post infection. Analysis of host and parasite miRNA abundance by qRT-PCR was extended to serum of patients from low and high infection sites in Zimbabwe and Uganda. The host-derived miRNAs failed to distinguish uninfected from infected individuals. However, analysis of three of the parasite-derived miRNAs (miR-277, miR-3479-3p and bantam) could detect infected individuals from low and high infection intensity sites with specificity/sensitivity values of 89%/80% and 80%/90%, respectively.

**Conclusions:**

This work identifies parasite-derived miRNAs as novel markers of *S. mansoni* infection in both mice and humans, with the potential to be used with existing techniques to improve *S. mansoni* diagnosis. In contrast, although host miRNAs are differentially expressed in the liver during infection their abundance levels in serum are variable in human patients and may be useful in cases of extreme pathology but likely hold limited value for detecting prevalence of infection.

## Author Summary

Schistosomiasis is a chronic disease caused by blood flukes that affects over 200 million people worldwide, of which 90% live in Sub-Saharan Africa. In the field setting schistosomiasis caused by *S. mansoni* is diagnosed by detection of parasite eggs in stool samples using microscopic techniques. Here we investigate the potential of microRNAs (miRNAs), a class of short noncoding RNAs, to act as biomarkers of *S. mansoni* infection. We have identified a specific subset of murine miRNAs whose expression is significantly altered in the liver between 6–12 weeks post infection. However their abundance in serum is not significantly different between naïve and *S. mansoni*-infected mice until twelve weeks post infection and they do not display consistent differential abundance in the serum of infected versus uninfected humans. In contrast, three parasite-derived miRNAs (miR-277, bantam and miR-3479-3p) were detected in the serum of infected mice and human patients and the combined detection of these miRNAs could distinguish *S. mansoni* infected from uninfected individuals from low and high infection intensity areas with 89%/80% or 80%/90% specificity/sensitivity, respectively. These results demonstrate that miRNAs of parasite origin are a new class of serum biomarker for detecting *S. mansoni* and likely other helminth infections.

## Introduction

Helminths are parasitic worms that infect a third of the world's population and cause a diverse range of health consequences leading to significant social and economical burdens [1], [2]. Schistosomiasis is a chronic disease caused by blood flukes of the genus *Schistosoma* that affects more than 200 million people worldwide and is second only to malaria as the most important lethal human parasitic disease in tropical and subtropical regions. Schistosomiasis is predominantly caused by hepatic *S. mansoni* and urogenital *S. haematobium* [3]. It is estimated that the mortality rates due to haematemesis (*S. mansoni*) and renal failure (*S. haematobium*) are around 130,000 and 150,000 per year respectively [4]. In addition, schistosomiasis is associated with anaemia, diarrhoea, under nutrition, chronic pain and exercise intolerance, which are estimated to contribute to 0.02–0.15 disability-adjusted life-years (DALY) [5].

Diagnosis of schistosome infection is crucial for patient management, evaluation of treatment efficiency, monitoring of disease transmission and success of control strategies, as recommended by the World Health Organization [6]. In the field, *S. mansoni* and other intestinal schistosomes are currently diagnosed through the detection of the parasite eggs in stool specimens using microscopic techniques such as Kato-Katz or ether-concentration [7]. While these techniques are relatively simple, inexpensive and specific, their major drawbacks include poor sensitivity in detecting low-intensity infections (for example, in children), their inability to detect pre-patent or single sex infection and their failure to detect infection in individuals where eggs are trapped in tissues and not excreted [8]. Available antibody-based assays are useful for diagnosis in some cases (e.g. foreign travellers) but they cannot differentiate past and active infection and can also cross-react with antigens from other helminths [9]. These assays therefore do not offer a definitive diagnosis in schistosome-endemic areas. Recent studies have shown success with point-of-care tests for Schistosoma circulating cathodic and anodic antigens (CCA and CAA, respectively) in serum and urine, which decrease rapidly after chemotherapy [8], [10], [11], [12], [13], [14] and these are now being further developed for use in the field [15]. Detection of schistosome DNA in urine and stool samples or plasma by the polymerase chain reaction (PCR) method is another strategy for routine diagnosis of infection that has shown promising results [16], [17], [18], [19], [20]. Here we examine whether microRNAs (miRNAs), which are extremely stable in serum and detectable by quantitative reverse transcription PCR (qRT-PCR) could provide an additional diagnostic tool for *S. mansoni* infection.

miRNAs are a class of naturally occurring small non-coding RNA produced from animal, plant and viral genomes [21]. They are incorporated into the RNA-induced silencing complex (RISC) and function by binding to messenger RNAs (mRNAs) and inhibiting translation and/or causing mRNA destabilization [22]. Depending on the genes they target, miRNAs have diverse functions inside cells, from regulation of developmental programming to viral-host interactions [23], [24], [25]. In the last 5 years, reports have shown that miRNAs circulate in serum in a cell-free form and many cell types secrete miRNAs through encapsulation within exosomes or in association with specific proteins [26], [27]. These extracellular RNAs have been implicated in cell-to-cell communication in a range of systems [28], [29], and have been shown to be extremely stable in body fluids [27], [30]. They have received extensive attention for their promise as biomarkers of disease, including cancer [31], [32], [33]. Extracellular miRNAs have also been shown to be altered in human serum and/or plasma in infection settings, including hepatitis C and hepatitis B infections [34], [35] as well as during pulmonary tuberculosis [36]. A recent report has also identified miRNAs derived from rice in human serum [37], sparking interest in foreign RNA in body fluids and its diagnostic potential.

Here we investigate the potential of miRNAs to act as tissue and serum biomarkers of experimental mouse and natural human *S. mansoni* infection. We demonstrate that several host miRNAs are dysregulated in the liver of mice during *S. mansoni* infection, but do not serve as reliable serum biomarkers of infection in humans. In contrast, we identify at least three parasite-derived miRNAs in the serum of mice infected with *S. mansoni* that are also detected in human patients and can distinguish ‘egg-negative’ from ‘egg-positive’ individuals with high specificity and sensitivity. We anticipate that parasite-derived miRNAs may provide a general platform for specific and non-invasive detection of active helminth infection.

## Methods

### Animals and *S. mansoni* infection

For miRNA array analysis of liver and Illumina sequencing of serum in experiment 1, 8–10 week old C57BL/6 mice were left uninfected or infected percutaneously with ∼180 *S. mansoni* cercariae, weighed regularly, and euthanized 7 weeks post infection. For the 12-week time course, 8–10 week old C57BL/6 mice were left uninfected or infected percutaneously with ∼80 cercariae, weighed regularly, and euthanized at 4, 6, 8 and 12 weeks post infection.

### Collection of blood and serum

Whole blood was drawn from mice by cardiac puncture. The needle was removed before emptying the syringe to avoid haemolysis and blood was allowed to sit for 1 h at RT to clot. Serum was separated by centrifugation at 2500 g for 15 min at 4°C, and the supernatant was collected into a new tube and spun at 10,000 g for 1 min to remove remaining cells. The resultant supernatant was transferred into a new tube and stored at −20°C prior to RNA extraction.

### Human serum samples

Human serum was screened retrospectively from samples obtained from schistosome endemic areas in Zimbabwe and Uganda. The study group included in this investigation (Zimbabwe) was part of a larger study on the molecular immunoepidemiology of human schistosomiasis (carried out between November 1999 and March 2000) and had not been included in the National Schistosome Control Programme and therefore had not received treatment for schistosomiasis or other helminth infections. After collection of all samples, all participants were offered anti-helminthic treatment with the recommended dose of praziquantel (40 mg/kg of body weight). The selection of samples for screening here was based on availability of sufficient serum for miRNA analysis, and comprised five ‘egg positive’ individuals with an average egg per gram in stool (epg) = 108, range: 39-277 and nine ‘egg-negative’ individuals (Table 1). According to the WHO's classification, Zimbabwe has high to moderate levels of *S. haemobium* infection (prevalence ranging from 10% to greater than 70%) but low *S. mansoni* prevalence (less than 10%). The selection of serum for screening from Piida (Uganda) participants was based on availability of sufficient material and comprised twenty individuals infected with *S. mansoni* with an average epg = 1117 (range: 105-4030) and ten egg-negative individuals. The study group included in this investigation were part of a larger study carried out in Butiaba village, adjacent to Lake Albert, Masindi district, Uganda in 1996, described further in [38]. The cohort had moderate to high *S. mansoni* infection intensities and prevalence of 91%. After collection of stool and serum samples all the study participants, irrespective of the infection status, received 2 doses of praziquantel, 40 mg/kg of body weight, 6 weeks apart. Efficacy of chemotherapy was assessed 6 weeks after each treatment.

**Table 1:** Top miRNAs dysregulated in the liver at 7 weeks post *S. mansoni* infection as determined by microarray analysis and showing results obtained by qRT-PCR (microarray: p<0.05, Fold change ≥2, qRT-PCR: t-test).

Up regulated miRNA				
miRNA	P value	Log2 (S.*mansoni* infected/Naïve ratio)	PCR Fold Change	PCR P value
miR-199-5p	0.0002	10.1	5.76	<0.0001
miR-199-3p	0.0001	6.4	5.7	<0.0001
miR-744	0.0002	5.3	1.2	NS
miR-214	0.0001	4.6	5.42	<0.0001
miR-210	0.0001	3.9	4.58	0.0001
miR-21	0.002	3.5	4.15	<0.0001

Down regulated miRNA				
miRNA	P value	Log2 (S.*mansoni* infected/Naïve ratio)	PCR Fold Change	PCR P value
miR-151-5p	0.024	−12.9	−1.42	0.0359
miR-9	0.031	−2.3	1.9	NS
miR-365	0.007	−2.2	−4.24	<0.0001
miR-192	0.006	−2.2	−3.35	<0.0001
miR-194	0.024	−2.0	−3.32	<0.0001
miR-122	0.028	−2.0	−3.2	<0.0001

### Ethics statement

Animal experiments were conducted under a Project License granted by the Home Office (United Kingdom), reference 60/4104, in accordance with local guidelines and approved by the Ethical Review Committee of the University of Edinburgh.

For the human serum samples collected in Chiredzi, permission to conduct the work in this province was obtained from the Provincial Medical Director. Ethical approval was received from the Medical Research Council of Zimbabwe (MRCZ). Only compliant participants were recruited into the study and they were free to drop out at any point during the study. At the beginning of the study, participants and their parents/guardians (in case of children) had the aims and procedures of the project explained fully in the local language, Shona, and oral consent (as was customary) was obtained from participants and parents/guardian before parasitology and blood samples were obtained. For the samples collected in Piida, ethical clearance was obtained from the Uganda National Council of Science and Technology (ethics committee for Vector Control Division, Ugandan Ministry of Health). The aims and procedures were explained to the local community at the start of the study and oral consent was obtained from all adults and from the parents/legal guardians of all children under 15 who were willing to participate. Due to cultural reasons and low levels of literacy, oral consent is deemed acceptable by the Ugandan Ministry of Health and was approved by the Uganda National Council of Science and Technology. Upon oral consent participants were enrolled in the study with a written record of their name, age, sex and case number, this served as both the record of oral consent and enrolment record.

### Parasitology and serum sample collection and processing

Participants in the Zimbabwe study were checked for both urogenital and intestinal schistosomiasis and for inclusion in this analysis had to be free of any soil-transmitted helminths and also free of *S. haematobium* infection to avoid cross reactivity between different helminth parasites. For the analysis of urogenital schistosome infection (*S. haematobium)* participants submitted three urine and three stool samples (over four consecutive days). 10 ml of each sample received was processed on the day of collection by a urine filtration method [39]. Stool samples were prepared and examined on the day of collection using the Kato-Katz faecal smear for detection of *S. mansoni* eggs and soil transmitted helminths; this was carried out by trained and experienced technical staff from the National Institute of Health Research [40]. A single slide for microscopic examination was prepared from each urine and stool sample. Serum was prepared from 10 ml venous blood collected from study participants, frozen at −20°C and afterwards stored at −80°C. Samples were transported frozen to Edinburgh and stored at −80°C prior to serological assays. Participants in the Ugandan study had duplicate 50 mg Kato-Katz slides prepared from 3 consecutive stool samples for detection of *S. mansoni* eggs, expressed as mean epg; this was carried out by trained and experienced technical staff from the Vector Control division at the Ministry of Health in Uganda. Serum was prepared from 10 ml venous blood samples, frozen at −20°C and transported to Cambridge for storage at −80°C prior to serological assays.

### Mouse serum RNA extraction

For the 12-week time course experiment, total RNA was extracted from serum using the miRVana PARIS extraction kit (Ambion), according to the manufacturer's protocol. In brief, 100 µl of serum was thawed on ice, mixed with an equal volume of 2× Denaturing Solution and kept on ice for 10 min. Samples were extracted with an equal volume of acid-phenol chloroform, vortexed for 30 s and centrifuged for 10 min at 10,000 g at RT. The aqueous phase was mixed with 1.25 volumes of 100% ethanol and added to the mirVana PARIS column. The column was washed and RNA eluted in 100 µl of 0.1 mM EDTA. RNA samples were stored at −20°C prior to further analysis. Extracted RNA was quantified by Qubit (Invitrogen).

### Human serum RNA extraction

Due to small volumes and low amounts of RNA in available human serum samples the extraction protocol was adjusted to result in more concentrated RNA. To do this, 50 µl of serum was thawed on ice, mixed with 50 µl of ddH_2_O and 100 µl of 2× Denaturing Solution (as supplied in the miRVana Paris kit) and kept on ice for 10 min. Samples were spiked with 10 fmoles of a synthetic RNA, Spike1: 5′-UGCUGAAUGCGUAGCUAUAAGC-3′ (IDT) and extracted with an equal volume of acid-phenol chloroform, vortexed for 30 s and centrifuged for 10 min at 10,000 g at RT. The aqueous phase was mixed with 1/10 volume of 3M sodium acetate, 10 µg of GlycoBlue (Ambion) and an equal volume of isopropanol. Samples were allowed to precipitate overnight at −20°C and were then centrifuged at >10,000 g at 4°C. Pellets were washed twice with 75% ethanol, air-dried and then resuspended in 25 µl of 0.1 mM EDTA. The total RNA concentration was below the limit of detection based on Qubit (Invitrogen).

### Liver RNA extraction

Liver tissue was immersed in RNA Later Solution (Ambion) overnight at 4°C prior to extraction using TRIzol Reagent (Invitrogen) according to the manufacturer's protocol. RNA was quantified by NanoDrop and integrity assessed by 10% PAGE or Bioanalyzer 2100. All RNA samples used in the microarray analysis had RIN >8.

### Reverse transcription & PCR

For reverse transcription of mouse serum samples, a fixed amount of extracted RNA (1.5 ng) was used as an input and 0.1 fmoles of a synthetic RNA, Spike2: 5′-CGUAUCGAGUGAUGUCACGUA-3′, was added at the RT step for normalization. For human serum samples, where the total RNA concentration was below the detection limit, a fixed volume of RNA (5 µl), corresponding to 10 µl of extracted serum, was used as the input and Spike1 (added at the time of purification) was used for normalization. For reverse transcription of RNA extracted from liver, 200 ng of total RNA was used in each reaction. Reverse transcription reactions were performed using the miScript System (Qiagen) according to the manufacturer's protocol. PCR was carried out with SYBR green real-time PCR assays (Qiagen) and miScript primers to detect mouse and human miRNAs, according to the manufacturer's protocol (Qiagen). Primers for *S. mansoni*-specific miRNAs and the synthetic spikes were used at 200 nM final concentration and were purchased from Invitrogen: miR-277, 5′-TAAATGCATTTTCTGGCCCG-3′, miR-2162-3p, 5′-TATTATGCAACGTTTCACTCT-3′, miR-3479-3p, 5′-TATTGCACTAACCTTCGCCTTG-3′, bantam, 5′- TGAGATCGCGATTAAAGCTGGT-3′, miR-2a-3p, 5′-TCACAGCCAGTATTGATGAAC-3′, miR-71a-3p, 5′- TGAAAGACGATGGTAGTGAGAT-3′, sma-miR-n1, 5′-AACTCAGTGGCCTATCGGT-3′, sma-miR-n2, 5′-TCAGCTGTGTTCATGTCTTCGA-3′, sma-miR-n3, 5′- TGGCGCTTAGTAGAATGTCACCG-3′, Spike1, 5′-TGCTGAATGCGTAGCTATAAGC-3′, Spike2, 5′-CGTATCGAGTGATGTCACGTA-3′. Data were collected on a Light Cycler 480 System (Roche) with the following temperature profile: pre-denaturation 15 min at 95°C followed by 50 cycles of denaturation for 15 s at 95°C, annealing for 30 s at 55°C, elongation for 30 s at 70°C. The efficiencies of pre-optimized miScript host miRNA probes (Qiagen) were measured from standard curves and ranged between 93–100% and the efficiency of custom probes ranged from 87–97%; both displayed homogenous melting curves and amplification products of the expected size, as described in [41], which were examined here by 6% TBE PAGE (data not shown). Two technical replicates were carried out for each biological replicate. Nuclease free water was used as a non-template control.

### miRNA array

For the array analysis, 1 µg of total RNA was labelled using the Hy3 power labeling kit (Exiqon) and hybridized to codelink slides printed with the miRCury 8.1 probe set as described elsewhere [42]. Hybridization and washing were carried out following the manufacturer's protocols (Exiqon). Background signal was subtracted from foreground signal and data were transformed to log-2 scale. Between-array normalization was carried out using global array percentiles (matching median of each array); triplicate probes were represented by the median for each array. Empirical Bayes moderated t statistic (eBayes) was used to test the null hypothesis of **“**no differential expression**”** between uninfected (n = 3) and infected (n = 3) liver samples. Since this array was only used as a filter for further validation, the p values were not adjusted for multiple testing. The Exiqon 8.1 arrays contained 384 probes specific for mouse miRNAs.

### qRT-PCR data analysis

For analysis of miRNAs in liver samples, the relative fold change between naïve and infected samples was calculated using the 2^−ΔΔCt^ method [43], normalized to miR-16; values for infected mice were compared to values for age-matched naïve controls and the median value for naïve mice was set to 1 for the purpose of calculating fold change. For serum miRNA data analysis, Ct values were “median-normalized” to synthetic RNA spike oligos as described previously [27]: relative change was calculated as 2^−Ctn^, where Ctn stands for normalized Ct values. The spike-in sequences did not match any known miRNA in miRBase and the primers for detecting these did not yield signals in serum by qRT-PCR, indicating that they do not cross-hybridize with mouse or human small RNAs (data not shown). Fold change was calculated as the ratio of the relative change value of the sample compared to an average of the relative change values of uninfected samples. For the cumulative analysis of miRNAs, the arithmetic mean of fold changes for miR-277, miR-3479-3p and bantam were used.

Statistical analysis of qRT-PCR data and receiver operator characteristic (ROC) curve analysis (95% confidence intervals) was performed with GraphPad Prism (Version 6) software. Two-way ANOVA followed by a Sidak multiple comparison test was used to calculate statistical differences for the mouse miRNA time course data from serum and liver. For the parasite miRNA serum time course, one-way ANOVA followed by Holm-Sidak multiple comparison was used. For analysis of the human serum samples, the Man-Whitney test was used and p-values of <0.05 were considered statistically significant. The measurement of miRNA levels by qRT-PCR was carried out by a trained doctoral student who was not blinded to the results of the infection status of each sample. Of the samples available for screening, none were excluded from the analysis.

### Illumina sequencing

For small RNA sequencing in experiment 1, total RNA was extracted from serum of 8 pooled *S. mansoni*-infected mice (Wk 7, 180 cercariae) and 8 uninfected age matched controls (400 µl total volume) according to the miRVana PARIS protocol (as described above). The small RNAs were size selected by 15% PAGE and prepared according to the Illumina small RNA Sample Preparation Kit version 1.5 and sequenced on the GAIIX. For small RNA sequencing in experiment 2, total RNA was extracted from serum of 3 pooled *S. mansoni* infected mice (Wk 8, 80 cercariae) and 3 uninfected age matched controls (300 µl total volume) according to the miRVana PARIS protocol. The library was prepared according to the TruSeq Small RNA protocol (without size-selecting small RNA) and sequenced on the HiSeq2.

Raw reads were obtained in fastq format and 3′ adapters trimmed using cutadapt, requiring at least a 6 bp match to the adapter sequence and a quality threshold of 20. Only reads that contained the adapter were retained; reads were subsequently collapsed on primary fasta sequence and only reads present at ≥2 copies were analyzed. Trimmed, collapsed reads ≥17 bp were then aligned to mouse (MM9) or *S. mansoni* genomes (V5.0) using BOWTIE version 0.12.5, requiring a perfect match to the full length of the sequence. Reads that mapped to either genome were then BLASTN aligned against the RFAM database [BLASTN parameters: -max_target_seqs 1 -outfmt ‘6 std qseq sseq’ -task blastn -word_size 6 -dust no] and categorized according to matches to Rfam class (e.g. rRNA, tRNA, etc.). Mouse reads without rfam similarities (other than miRNAs) were aligned to mature miRNAs in miRBase version 19. Some trimmed miRNA reads aligned to more than one family member: the assignment of these ambiguous reads is designated with “X” in Table S3. RNAs that aligned to the *S. mansoni* genome and did not show RFAM similarities were passed to mirDeep2.0.0.5 using platyhelminth miRNAs from miRBase 19 as guides (Table S4); reads mapping to known miRNAs from miRBase were identified regardless of miRdeep score, for prediction of novel miRNAs a cut-off value of 0 was used for reporting in Table 1.

## Results

### Specific host miRNAs are significantly dysregulated in the liver of mice at 7 weeks post *S. mansoni* infection

miRNAs are dysregulated in most disease contexts and play important roles in mediating how cells respond to insult and infection (reviewed in [44]). Approximately 4–6 weeks after *S. mansoni* infection, mature female parasites produce eggs, some of which are carried by the blood-flow to the liver where they become trapped [45]. The host immune response induced by the presence of the egg antigens leads to the formation of granulomatous lesions, which are composed of immune cells and collagen fibres [46] and result in fibrosis and associated pathology. In order to identify miRNAs associated with *S. mansoni*-induced liver pathology, and to prioritise candidates for further screening as biomarkers, we first compared expression profiles of miRNAs in livers of naïve mice or mice that were infected with *S. mansoni* at a high dose (∼180 cercariae). Tissues were collected at 7 weeks post infection, at which time substantial granulomas were observed (data not shown). A total of 33 mouse miRNAs were differentially expressed: 26 miRNAs were up-regulated and 7 miRNAs were down-regulated in infected mice, based on a fold change cut-off of ≥2 and p value cut off <0.05 (Table S1, Fig. S1). The miRNAs that displayed the largest differential expression included miR-199a and miR-214, which are known to be altered in liver fibrosis caused by hepatitis C infection or induced by carbon tetrachloride [47], [48]. Among the down regulated miRNAs was the liver-enriched miR-122, which is dysregulated during hepatitis C infection, acetaminophen overdose and hepatocellular carcinoma and is involved in lipid metabolism [49], [50].

For validation of the microarray results, the miRNAs that displayed the largest fold change were quantified by qRT-PCR and normalized to miR-16 (a total of 6 up-regulated miRNAs and 6 down-regulated miRNAs were examined). Consistent with the array results, there was an increase in miR-199-5p, miR-199-3p, miR-214, miR-21, miR-210, and a reduction of miR-192, miR-194, miR-365, miR-122 and miR-151 in the liver tissue of *S. mansoni* infected mice as compared to naïve mice; miR-9 and miR-744 did not display differential expression and were not analysed further (Table 1). All of these miRNAs are perfectly conserved in mouse and human.

### Temporal expression analysis of miR-199, miR-214, miR-21, miR-210, miR-122, miR-192 and miR-194 in the liver during *S. mansoni* infection

**Figure 1:**
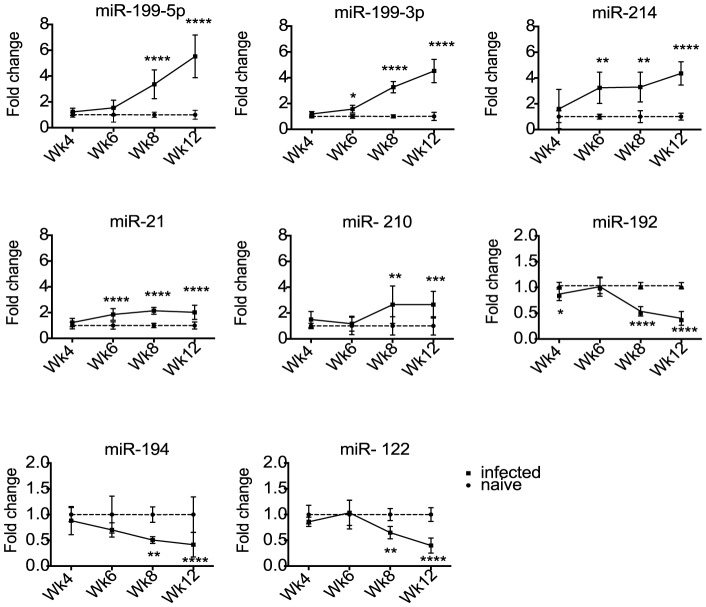
Differential expression of host miRNAs in the livers of mice at 4–12 weeks post infection with *S. mansoni*. miRNAs were quantified by qRT-PCR, normalized to miR-16 and fold changes calculated as the ratio of values from infected versus naïve mice (*p<0.05, **p<0.01, ***p<0.001, ****p<0.0001).

Between weeks 6 and 12, female parasites continue to produce ∼300 eggs per day [51], resulting in an increase in the number of granulomas in the liver and the development of fibrosis [45]. For the 10 miRNAs validated to be differentially expressed at 7 weeks post infection, we next examined their temporal expression between 4–12 weeks post infection using a lower parasite dose (80 cercariae). All data from infected mice were compared to age-matched naive mice. To account for differences in RNA extraction or qRT-PCR efficiency, the data were normalised to miR-16, which displayed stable expression in the liver during infection (Fig. S2). Of the 10 miRNAs examined, all except miR-365 and miR-151 were differentially expressed between naïve and infected mice by 6–8 weeks post infection (Fig. 1, Table S2). This timing correlated with the deposition of eggs in the liver, which were detected by 6 weeks post infection and increased by 8 and 12 weeks post infection (Fig. S3). Our results suggest that these cellular miRNAs represent tissue biomarkers of infection that may play a role in the development and progression of liver fibrosis induced by *S. mansoni* egg deposition.

### Temporal host miRNA dysregulation in the liver during infection is not reflected in serum

**Figure 2:**
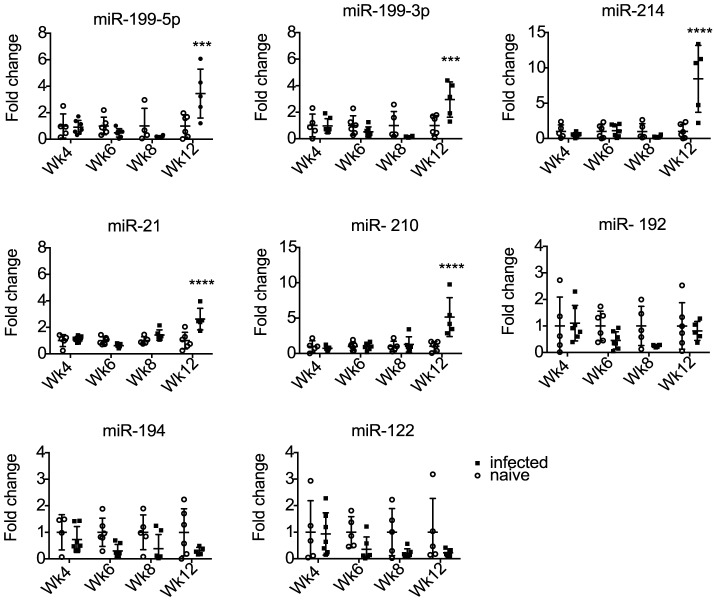
Differential abundance of host miRNAs in mouse serum during *S. mansoni* infection. miRNAs were quantified by qRT-PCR and normalized to a synthetic RNA spike-in. Each symbol represents data from one individual mouse. Fold changes are defined as the ratio of abundance in infected versus naïve serum; the signal from naïve was set as 1. (***p<0.001, ****p<0.0001).

Several studies in non-helminth systems have shown that liver-derived miRNAs are detectable in serum and can be used as biomarkers in disease states [52], [53], [54], [55], [56]. We therefore examined whether the murine miRNAs that are altered in liver tissue are similarly altered in serum during *S. mansoni* infection. qRT-PCR was used to measure miRNA levels in the serum of mice infected with *S. mansoni* over the 12 week time course. The methodology of miRNA analysis in body fluids is not well standardized, and it is still not clear which small RNAs are appropriate for normalization [57], [58], [59], [60]. Here we used a constant amount of total RNA in the reverse transcription reaction. To account for any variation in qRT-PCR efficiency due to contaminants, miRNA levels were normalized to a synthetic RNA oligo (“spike-in”) that was included at the reverse-transcription step. The ratio of miRNA levels in infected versus age-matched naïve mice was quantified at each time point and plotted as fold change (Fig. 2). Compared to the analysis of liver samples, there is more variation in the serum miRNA levels between biological replicates. As shown in Fig. 2, the levels of miR-192, miR-194 and miR-122 in serum do not change between 4–12 weeks post infection, whereas five of the miRNAs that are up-regulated in the liver are also significantly elevated in serum at 12 weeks post infection (p<0.05), ranging from 2.6 fold (miR-21) to 4.7 fold (miR-214) (Table S2). These five host miRNAs represent potential serum biomarkers of *S. mansoni* infection. However, since their levels do not change until 12 weeks post infection in mice, they may only be useful in cases of more advanced pathology.

### Small RNA sequencing reveals the presence of *S. mansoni*-derived miRNAs in the serum of infected mice

**Figure 3:**
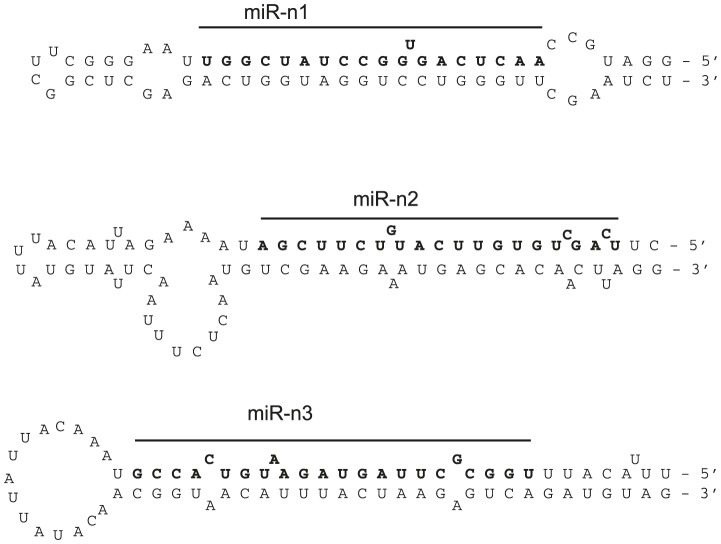
Predicted stem-loop structures of the *S. mansoni* miRNAs identified in this study. The mature sequence that was identified in serum is shown in bold.

Multiple cell types release or secrete miRNAs into the circulation [59], [61] and it is possible that other miRNAs, beyond those differentially expressed in the liver, might represent biomarkers of infection. To examine this in an unbiased fashion, small RNAs from serum of naïve mice or mice infected with *S. mansoni* were sequenced using the Illumina platform. Small RNA libraries were prepared in two independent experiments, using two different preparation methods (Methods). In both experiments, the majority of small RNA reads aligned to the mouse genome (ranging from 63–71%, Table 2). A small percentage of reads unambiguously aligned to the *S. mansoni* genome in infected mice (0.04–0.14%); <0.01% of reads mapped to the *S. mansoni* genome in naïve samples. Interestingly, the majority of reads in all samples were derived from tRNAs: 92–98% of the reads that mapped to the mouse genome and 42–100% of reads that mapped to the *S. mansoni* draft genome (Table 2). It is important to note, however, that due to the sequence similarity of the tRNAs between species and the scope for post-transcriptional editing, we cannot at this point definitively determine the organism from which these derive. Only 1–6% of the reads that mapped to the mouse genome were miRNAs (Table 2, listed in Table S3). There was no correlation between differences in host miRNA levels in naive and infected mice from the two experiments (data not shown), this could relate to the different doses, although it is not possible to derive statistical conclusions from these data due to low read numbers. A total of 78 and 29 mature miRNAs are known to be encoded by the trematodes *S. japonicum* and *S. mansoni*, respectively [62], [63], [64], [65], and further miRNAs have been predicted [62]. Analysis of the reads that mapped to the *S. mansoni* draft genome using the miRdeep2 program [66] identified at least 11 miRNAs from infected samples: 8 of these were positively identified based on identity to known miRNAs in *S. mansoni* and/or S. *japonicum* (Table 3) [64], [65] and 3 are predicted by miRdeep (here named sma-miR-n1, sma-miR-n2 and sma-miR-n3). The predicted stem-loop structures for the putative pre-miRNAs are shown in Fig. 3; since the depth of coverage of *S. mansoni* reads in this study was very low and reads did not map to both arms of the hairpin, these cannot yet be considered prototypical miRNAs. However, sma-miR-n3 shares a seed site with *Schmidtea mediterranea* miR-2160 and was annotated as sma-miR-8437 in a study of *S. mansoni* miRNAs published after submission of this manuscript [67]. Additional analysis of the datasets allowing 1 mismatch to the *S. mansoni* draft genome identified one further miRNA from infected but not naïve serum: miR-277, which is identical to miR-277 in *S. japonicum* and *Echinococcus granulosus* but has a C→T mutation at position 17 in relation to the *S. mansoni* draft genome (Table 3). Other reads from infected samples that aligned to *S. mansoni* but did not make the miRdeep2 cut-offs are provided in Table S4. It is possible that some of these less abundant sequences derive from real miRNAs but the coverage in these studies is not sufficient to determine this.

**Table 2:** Small RNA classification in serum of naïve and infected mice.

	Illumina Small RNA (Experiment 1)		TruSeq (Experiment 2)	
	Naïve	Infected	Naïve	Infected
		7 weeks		8 weeks
		180 cercariae		80 cercariae
Trimmed reads	19,992,215	19,687,439	5,360,110	6,975,834
Mouse genome match	12,864,294	12,771,443	3,786,803	4,621,905
**Unambiguous**	**12,841,090(64.2%)**	**12,430,681 (63.1%)**	**3,779,361 (70.5%)**	**4,613,742 (66.1%)**
rRNA	6493 (0.05%)	51173 (0.4%)	377 (0.01%)	6499 (0.14%)
tRNA	12,615,823 (98.2%)	11,464,782 (92.2%)	3,736,007 (98.9%)	4,409,765 (95.6%)
other rfam	7579 (0.06%)	14563 (0.012%)	514 (0.014%)	1758 (0.04%)
miRNA	128,222(1.0%)	772,150 (6.2%)	36,615 (1.0%)	158,069 (3.4%)
Uncharacterized	82973 (0.6%)	128013 (1%)	5848 (0.16%)	37651 (0.8%)
*S.mansoni* genome match				
**Unambiguous**	**1,683 (<0.01%)**	**27,347(0.14%)**	**529 (<0.01%)**	**2,914 (0.04%)**
rRNA	272 (16.2%)	2828 (10.3%)	0	182 (6.2%)
tRNA	1,236 (73.4%)	20,191 (73.8%)	529 (100%)	1,231 (42%)
other rfam	52 (3.1%)	117 (0.4%)	0	22 (0.75%)
miRNA	2 (0.12%)	148 (0.54%)^1^	0	25 (0.9%)
Uncharacterized	121 (7.2%)	4,063 (15%)	0	1,454 (49.9%)

1 This does not include 42 reads identical to sja-miR-277 that contain 1 nucleotide mismatch to *S. mansoni* draft genome.

**Table 3:** Parasite-miRNAs in serum of mice infected with *S. mansoni* identified by deep-sequencing.

Name	miRNA sequence (5′-3′)	Precursor Location	Reads^2^
sma-miR-n2	UCAGCUGUGUUCAUGUCUUCGA	S_mansoni.SC_0170:285549..285631:-	66
sja-miR-277^1^	UAAAUGCAUUUUCUGGCCCGUA	Inferred from sja-miR-277 in miRBase	42
sma-bantam	UGAGAUCGCGAUUAAAGCUGGU	S_mansoni.SC_0137:369450..369509:+	37
sja-miR-2162-3p	UAUUAUGCAACGUUUCACUCU	S_mansoni.SC_0049:36195..36251:+	29
sma-miR-3479-3p	UAUUGCACUAACCUUCGCCUUG	S_mansoni.Chr_4.unplaced.SC_0032:1561293..1561349:-	16
sma-miR-n1	AACUCAGUGGCCUAUCGGU	S_mansoni.Chr_1:18109172..18109229:-	9
sma-miR-n3	UGGCGCUUAGUAGAAUGUCACCG	S_mansoni.Chr_3:22962153..22962218:+	7
sma-miR-10-5p	AACCCUGUAGACCCGAGUUUGG	S_mansoni.Chr_4:19959278..19959336:-	4
sma-miR-2a-3p	UCACAGCCAGUAUUGAUGAAC	S_mansoni.Chr_W:22875762..22875816:+	3
sma-let-7-3p^3^	CAUACAACCGACUGGCUUUCC	S_mansoni.Chr_7:5118795..5118860:+	2
sma-miR-71a-3p	UGAAAGACGAUGGUAGUGAGAU	S_mansoni.Chr_W:22875670..22875724:+	2

1 The nucleotide which does not match the *S. mansoni* draft genome is shown in bold;

2 Reads in combined infected samples (compared to a combined total of 930,209 mouse miRNAs in the same samples, Table 1);

3 The 3p arm of sma-let-7 is not annotated in mirbase.

### *S. mansoni* miRNAs are present in mouse serum as early as 8 weeks post infection

**Figure 4:**
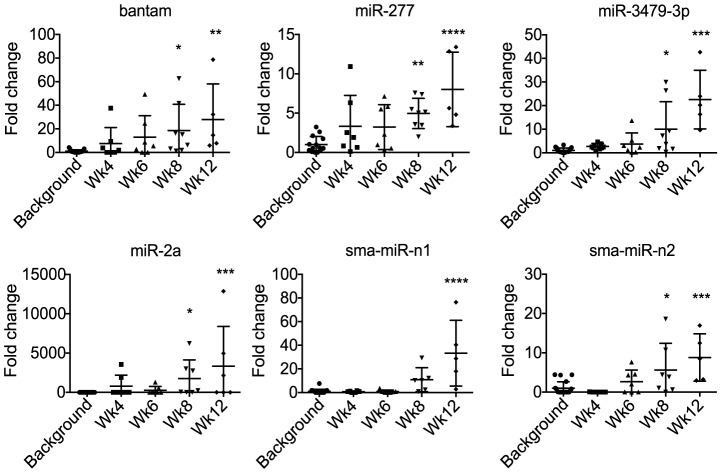
Detection of parasite-derived miRNAs in mouse serum during *S. mansoni* infection. miRNAs were quantified by qRT-PCR, normalized to a synthetic RNA spike-in and fold change calculated as the ratio of abundance in infected serum compared to the background abundance level detected in naïve serum, which represents the noise in the assay likely derived from cross-hybridization with endogenous small RNAs (*p<0.05, **p<0.01, ***p<0.001, ****p<0.0001).

To determine the kinetic profile of parasite miRNAs in serum during *S. mansoni* infection, qRT-PCR analysis was carried out as described above, using primers specific for 9 of the 11 parasite miRNAs. The other 2 miRNAs, sma-miR-10-5p and sma-let-7-3p, were excluded from analysis because they are highly similar to homologous mouse miRNAs that are present at >100 fold higher read frequencies (Table S3). Importantly, most miRNAs in helminth parasites have evolved after the last common ancestor with their vertebrate hosts and are therefore distinguishable in sequence, however several miRNAs are perfectly conserved across animals or highly similar in sequence [63]. Several of the parasite-specific probes showed a signal in the serum of naïve mice, which is presumably due to cross-hybridization with endogenous small RNAs. In cases where no signal was observed in naïve mice, the maximum cycle value of 50 was set as background for the purpose of calculating signal over noise (which we interchange here with “fold change”). Six of the nine parasite miRNA probes tested (miR-277, bantam, miR-3479-3p, miR-2a-3p, miR-n1, miR-n2) showed a statistically significant signal over noise at 8 or 12 weeks post infection (Fig. 4, p<0.05); the three miRNAs that were not reliably detected (miR-n3, miR-71a-3p, miR-2162-3p) were not analysed further. The average signal over noise ratios for each probe during the time course of infection are provided in Table S5 and range from 4.2 to >3,000.

### Three *S. mansoni*-derived miRNAs are detected in human serum and distinguish egg-negative from egg-positive individuals

**Figure 5:**
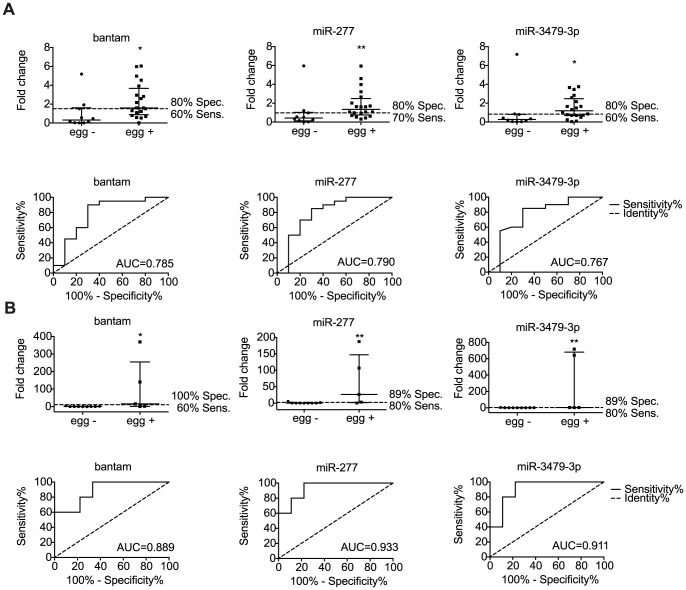
Discrimination between the *S. mansoni* infected and uninfected individuals using parasite-specific miRNA detection. miRNAs were quantified by qRT-PCR, normalized to a synthetic RNA spike-in and fold changes calculated as the ratio of infected to uninfected (median with interquartile range indicated). Piida- panel A, Chiredzi- panel B. Specificity and sensitivity and ROC curves with AUC are indicated. (*p<0.05, **p<0.01).

**Figure 6:**
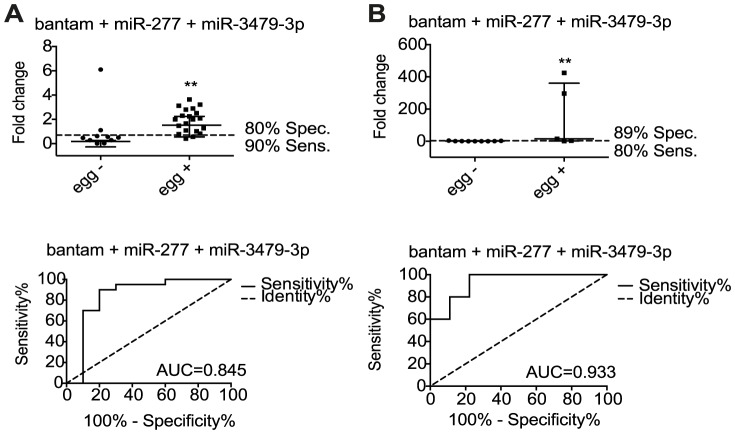
Discrimination between the *S. mansoni* infected and uninfected individuals using the combined data for 3 parasite miRNAs. Fold-changes (median with interquartile range indicated) and ROC curves for bantam, miR-277 and miR-3479-3p (Piida- panel A, Chiredzi-panel B) (**p<0.01).

Based on the results described above, we extended our analyses to human patients, using the 6 parasite miRNAs and 5 mouse miRNAs that displayed differential abundance in serum of infected compared to naïve mice. Serum samples from two field sites were examined: an area of high infection in the Piida community of Uganda and an area of low infection in the Chiredzi community of Zimbabwe. The high infection samples were collected from mixed-age participants from Piida diagnosed as *S. mansoni* infected, termed ‘egg positive’ and compared to volunteers from the same community with undetectable parasite eggs in the stool, termed ‘egg-negative’. The low infection samples were collected from children in Chiredzi diagnosed with *S. mansoni* and compared to age matched participants with undetectable eggs by standard stool examination methods. Demographic data of individuals are provided in Tables 4 and 5. The signal over noise was calculated as described above, using synthetic spike-ins for normalization. miR-n1, miR-n2 and miR-2a-3p were below the detection limit (Ct = 50) in both ‘egg-positive’ and ‘egg-negative’ samples. The 5 host miRNAs were detectable in serum (miR-21, miR-199-3p, miR-199-5p, miR-210, miR-214) but showed variable abundance and failed to differentiate ‘egg-positive’ and ‘egg-negative’ participants (Fig. S4). In contrast, three out of the six parasite miRNAs (bantam, miR-277 and miR-3479-3p) displayed a significant signal over noise level in the serum of *S. mansoni* infected individuals ‘egg-positive’ from both high (Fig. 5A) and low (Fig. 5B) infection endemic areas, compared to the ‘egg-negative’ participants from the same communities (p<0.05, Mann – Whitney test). Data presented using ROC curves show that the single parasite miRNAs discriminated between *S. mansoni* ‘egg-negative’ and ‘egg-positive’ with an area under the curve (AUC) of 0.785, 0.790, 0.768 for bantam, miR-277 and miR-3479-3p, respectively, in the individuals from Uganda (Fig. 5A) and 0.889, 0.933, 0.911 in the individuals from Zimbabwe (Fig. 5B). Using optimal cut-off points, this translates to detection of *S. mansoni* infected individuals with specificity/sensitivity of 80%/60%, 80%/70% and 80%/60%, in the patients from Uganda (Fig. 5A) and specificity/sensitivity of 100%/60%, 89%/80% and 89%/80% respectively in the patients from Zimbabwe (Fig. 5B). A repeated measurement of the parasite miRNA levels in the same samples displayed Pearson correlation values between 0.86 to 0.95 and comparable specificity/sensitivity values (Table S6). When combining the data for all three of the miRNAs into a cumulative value, the AUC increased to 0.845 and 0.933 for each cohort of participants. This resulted in improved specificity/sensitivity in detection for samples from Uganda (80%/90%) using a fold change cut-off of 1.189 (Fig. 6). This approach is very similar to a recent report that uses a “miRNA score” based on cumulative normalized signals of miRNAs [68]; analysis of our data based on cumulative fold change or cumulative normalized signals yields very similar results (Fig. S5). These results show that combining data for bantam, miR-277 and miR-3479-3p may improve sensitivity of *S. mansoni* diagnosis compared to analysis of individual miRNAs.

**Table 4:** Piida study participants.

Characteristic	Egg-negative (n = 10)	Egg-positive (n = 20)
Age (range)	33.1 (17–48)	24.2 (7–56)
Sex (F/M)	5/5	9/11
*S.mansoni* status (epg & range)	0	1117 (105–4030)

**Table 5:** Chiredzi study participants.

Characteristic	Egg-negative (n = 9)	Egg-positive (n = 5)
Age (range)	10.67 (8–11)	10 (9–11)
Sex (M/F)	6/3	2/3
*S.mansoni* status (epg & range)	0	108 (39–277)

## Discussion

The recent evidence that miRNAs can be released into circulation from mammalian cells and tissues has stimulated extensive interest in the potential use of these molecules as non-invasive biomarkers [31], [32], [57]. MiRNA-based diagnostics are being developed for a number of diseases and although qRT-PCR is the most common detection method at present, there is extensive interest in improving and diversifying detection technologies, which may provide more field-friendly tools. Since miRNAs have been shown to be extremely stable in body fluids [27], [30], [69], we anticipate that these nucleic acids could be particularly useful as diagnostics in field settings where collection and storage conditions can be difficult to control. Here we find that miRNAs derived from the helminth parasite *S. mansoni* are present in infected mouse and human serum and offer advantages over endogenous miRNAs as biomarkers of infection. Specifically, we find 9 known miRNAs and at least 2 novel putative miRNAs derived from *S. mansoni* that are present in the serum of infected mice. The read counts of some of these are very low (between 2–66 reads per 930,209 mouse miRNAs reads) and will require further validation with better coverage. However, we show that three of these miRNAs (bantam, miR-277, mirR-3479-3p) can be detected in human serum from schistosome endemic areas. These represent a direct marker for infection and may also provide an indirect marker for the pathology induced by infection. Notably, a study published after submission of this manuscript identified 5 miRNAs derived from *S. japonicum* in the plasma of infected rabbits and 3 of these are identical or homologous to those identified here: bantam, miR-3479-3p and miR-10-5p [70], providing independent validation for the presence of trematode miRNAs in the serum of infected animals. Here we demonstrate small RNAs derived from a helminth parasite are also present in patient serum, and provides a starting point for developing more field-friendly methods for their detection. This work also extends a burgeoning area of research detailing “foreign” small RNAs in body fluids. Zhang et al., (2012) recently reported that the plant miRNA, miR-168a, is present in human and animal serum and demonstrated that this derives from a rice diet [37]. Wang et al., (2012) recently reported that a wide range of exogenous RNAs can be found in human plasma, including small RNAs derived from bacteria and fungi [71]. From our results is not yet clear whether the miRNAs identified in serum are actively secreted from the parasite and how they are stabilized, since serum contains high amounts of RNases [72], [73]. From the time course analysis presented here, schistosome-derived small RNAs were reliably detected in serum by 8 weeks post infection, after deposition of the eggs in the liver (Fig. 4 and Fig. S4). At present, we cannot determine whether these RNAs derive from the adult worms or the eggs; further *in vitro* and *in vivo* studies will shed light on this issue, which is relevant to diagnostic applications, for example the capacity to detect pre-patent infection.

It is intriguing to think that existence of these miRNAs outside the parasite has a function, but this is beyond the scope of the current analysis. Given the short size of miRNAs and the fact that they do not require perfect complementarity with their targets, one could predict hundreds of possible targets in the host. Interestingly, Xue et al. (2008) showed that three of the miRNAs that we find in serum (sja-bantam, sja-miR-71 and sja-let-7) are expressed during all the stages of parasite development but are enriched in the cercariae, suggesting that they may be important during the initial stages of schistosome infection [65]. The bantam miRNA has been implicated in regulating organ growth in response to environmental conditions in *Drosophila* as well as *C. elegans* [74], [75] but functional homologues of bantam do not exist in mammals.

An initial objective in this study was to identify host miRNAs that may be involved in liver pathology associated with *S. mansoni* infection and to then determine whether these hold any diagnostic value. A number of reports have demonstrated an increase in miR-122 and miR-192 in plasma or serum upon viral infection as well as chemically induced liver disease [54], [56]. However, according to our analysis, although miR-192, miR-122 and miR-194 were down-regulated in the liver during infection, their levels in serum did not change significantly (Fig. 1–2). In contrast, the miRNAs up-regulated in the liver (miR-199-3p, miR-199-5p, miR-21, miR-214 and miR-210) showed significantly higher levels in mouse serum at 12 weeks post infection (Fig. 2), however these failed to differentiate *S. mansoni* infected from uninfected humans (Fig. S4). It should be noted that in addition to significant liver disease, pulmonary and intestinal complications can also occur during *S. mansoni* infection that could also contribute to serum miRNA levels [45], [76]. Related to this, a possible limitation in the use of endogenous miRNAs as biomarkers is the fact that their differential abundance in serum can derive from multiple cell types and can also be attributed to unrelated conditions [59]. Although the diagnostic value of these host miRNAs in serum is therefore not obvious, our work provides a foundation for further research into the functional role of these miRNAs in *S. mansoni* pathogenesis. Interestingly, our results do not overlap with those reported by Han et al., (2013) who examined changes in host miRNA levels in the liver of BALB/c mice during *S. japonicum* infection. We assume this may be due to the very early time point (10 days post infection) used in their study. Indeed, it is likely that the host miRNA changes we observe are primarily related to the liver pathology and/or the immune response initiated by the parasite eggs trapped in the liver, rather than the initial host immune response to the schistosomula. Several of the miRNAs we identify as differentially expressed are already known to be associated with liver fibrosis or disease in other, non-helminth, settings [49], [50], [77], [78]. The functional role of the miRNAs in the pathology induced by *S. mansoni* infection remains to be determined. Future work in this area will shed light on the molecular basis of pathology and may offer innovative new therapeutic strategies.

Importantly, we report here that parasite-derived miRNAs can be detected in human serum and can distinguish ‘egg-negative’ from ‘egg-positive’ individuals in areas of both low and high infection intensity. By combining data for miR-277, miR-3479-3p and bantam we detected infection with a sensitivity of 80–90% and specificity of 80–89%. We anticipate that this may be improved further by optimizing isolation protocols, probe design and more robust methods for normalizing the data, for example to identify appropriate endogenous small RNAs that could be used as controls, rather than synthetic spike-ins [79]. The main limitation of miRNA detection in serum appears to be cross-hybridization of the probes with endogenous small RNAs, which influences the signal to noise ratios. In particular, we report the finding that the majority of small RNAs in mouse serum are derived from tRNAs (Table 2), consistent with a recent study [80]. It is possible that depletion of these tRNAs prior to qRT-PCR may improve the specificity or sensitivity of miRNA detection; this requires further investigation. In addition, one of the parasite-derived miRNAs identified by sequencing, miR-2162-3p could not be validated by qRT-PCR, likely owing to its low GC content (33%). Optimization of probe design for the parasite-derived miRNAs may also greatly increase their diagnostic utility in larger scale studies in human patients. On this note, the work presented here was performed on a relatively small number of individuals. Following further optimization of the extraction methods and probe design to minimize cross hybridization and sensitivity, larger studies will be important to assess the full potential of the proposed miRNA biomarkers, including positive and negative predictive values in comparison to existing techniques. It will also be of interest to determine the origin of these parasite-derived miRNAs and examine their abundance levels in response to treatment. We anticipate that the parasite miRNAs in serum could complement or transform existing diagnostic strategies and may serve as a platform for detecting a range of helminth infections.

## Supporting Information

Figure S1 Volcano plot of miRNA microarray data comparing miRNA expression levels in mice infected with *S. mansoni* compared to naive mice. The horizontal line indicates a p-value cut-off of 0.05 and vertical lines indicate fold change thresholds of 2.
(EPS)

Figure S2 Expression of miR-16 in liver and serum over the time course of *S. mansoni* infection. miRNAs were quantified by qRT-PCR, relative change was calculated as 2^−Ct^ (for liver samples) or 2^−Ctn^,(for serum samples) and fold changes were calculated as infected to naïve ratio.
(EPS)

Figure S3 *S. mansoni* egg counts in the livers of mice over the course of infection.
(EPS)

Figure S4 Abundance of host miRNAs in serum of *S. mansoni* egg-positive and egg-negative individuals. miRNAs were quantified by qRT-PCR, normalized to a synthetic RNA spike-in and fold changes calculated as the ratio of infected to uninfected individuals. Piida- panel A, Chiredzi- panel B.
(EPS)

Figure S5 Discrimination between the *S. mansoni* infected and uninfected patients using the miRNA-score. Dot plot comparing cumulative (bantam, miR-277 and miR-3479-3p) miRNA-score between *S. mansoni* egg positive and negative participants (median with interquartile range indicated) and ROC curves (Piida- panel A, Chiredzi-panel B) (**p<0.01).
(EPS)

Table S1 miRNAs that are dysregulated in the liver upon *S.mansoni* infection as determined by microarray analysis (p<0.05, fold change ≥2).
(DOCX)

Table S2 Relative expression of miRNAs in the liver and serum during the time course of *S.mansoni* infection based on qRT-PCR analysis, normalized to values in naïve mice.
(DOCX)

Table S3 Tabulated mouse miRNA reads identified in serum of naïve and infected mice. The “X” denotes the miRNAs who were assigned reads that could have derived from one of multiple miRNA family members (e.g. in the case of shortened reads).
(XLSX)

Table S4 Prediction of known or novel *S. mansoni* miRNAs in mouse serum based on miRdeep2. This table shows all predictions (only predictions with a miRdeep2 score >0 are included in the text). Read assignments are: uninfected experiment 1 (R1N), infected experiment 1 (R1I), uninfected experiment 2 (607), infected experiment 2 (608 or 609). The number of reads in that sample is listed after the “x”.
(TXT)

Table S5 Relative expression of parasite miRNAs in serum during the time course of *S.mansoni* infection, based on qRT-PCR analysis, normalized to values in naïve mice.
(DOCX)

Table S6 miRNA detection values in Chiredzi and Uganda samples. Fold change is defined as the abundance value in each sample compared to the median of values obtained in uninfected individuals. Repeated miRNA measurements from Chiredzi samples are shown (there was not enough material to repeat measurement in Uganda samples).
(XLSX)

Table S7 Ct values obtained for qRT-PCR.
(XLSX)

Checklist S1 STARD Checklist.
(DOC)
