# Supplementary material for: Parasite-Derived MicroRNAs in Host Serum As Novel Biomarkers of Helminth Infection
Source: PLoS Negl Trop Dis. 2014 Feb 20;8(2):e2701. doi: 10.1371/journal.pntd.0002701 (PMC3930507; doi:10.1371/journal.pntd.0002701)
Supplement: Table S1 — miRNAs that are dysregulated in the liver upon S.mansoni infection as determined by microarray analysis (p<0.05, fold change ≥2). (DOCX) [file pntd.0002701.s006.docx]

Table S1: miRNAs that are dysregulated in the liver upon *S.mansoni* infection as determined by microarray analysis (p<0.05, Fold change ≥2)

| name | P value | FC |
| --- | --- | --- |
| mmu-miR-199b*/miR-199a-5p | 0.0002 | 10.1 |
| mmu-miR-199a-3p/mmu-miR-199b | 0.0001 | 6.4 |
| mmu-miR-744 | 0.0002 | 5.3 |
| mmu-miR-292-5p | 0.0121 | 5.1 |
| mmu-miR-214 | 0.0001 | 4.6 |
| mmu-miR-210 | 0.0001 | 3.9 |
| mmu-miR-541 | 0.0467 | 3.8 |
| mmu-miR-21 | 0.0023 | 3.5 |
| mmu-miR-15b | 0.0018 | 3.2 |
| mmu-miR-181a | 0.0004 | 3.1 |
| mmu-miR-503 | 0.0002 | 3.0 |
| mmu-miR-689 | 0.0008 | 3.0 |
| mmu-miR-34a | 0.0018 | 2.9 |
| mmu-miR-497 | 0.0021 | 2.8 |
| mmu-miR-21* | 0.0012 | 2.8 |
| mmu-miR-330* | 0.0033 | 2.8 |
| mmu-miR-322 | 0.0011 | 2.7 |
| mmu-miR-500 | 0.0008 | 2.6 |
| mmu-miR-146b | 0.0041 | 2.5 |
| mmu-miR-155 | 0.0008 | 2.4 |
| mmu-miR-501-5p | 0.0077 | 2.3 |
| mmu-miR-143 | 0.0021 | 2.3 |
| mmu-miR-342-3p | 0.0072 | 2.3 |
| mmu-miR-145 | 0.0019 | 2.3 |
| mmu-miR-147 | 0.0090 | 2.2 |
| mmu-miR-335-5p | 0.0183 | 2.0 |
| mmu-miR-151-5p | 0.0241 | -12.9 |
| mmu-miR-192 | 0.0478 | -2.5 |
| mmu-miR-9 | 0.0309 | -2.3 |
| mmu-miR-365 | 0.0074 | -2.2 |
| mmu-miR-192 | 0.0064 | -2.2 |
| mmu-miR-194 | 0.0243 | -2.0 |
| mmu-miR-122 | 0.0289 | -2.0 |
