# Supplementary material for: Parasite-Derived MicroRNAs in Host Serum As Novel Biomarkers of Helminth Infection
Source: PLoS Negl Trop Dis. 2014 Feb 20;8(2):e2701. doi: 10.1371/journal.pntd.0002701 (PMC3930507; doi:10.1371/journal.pntd.0002701)
Supplement: Table S2 — Relative expression of miRNAs in the liver and serum during the time course of S.mansoni infection based on qRT-PCR analysis, normalized to values in naïve mice. (DOCX) [file pntd.0002701.s007.docx]

Table S2: Relative expression of miRNAs in the liver and serum during the time course of *S.mansoni* infection based on qRT-PCR analysis, normalized to values in naïve mice.

| ttest |  | Liver Fold change | Sidak's multiple comparison test | Serum Fold Change | Sidak's multiple comparison test |
| --- | --- | --- | --- | --- | --- |
| miR-199-5p | Wk4 | 1.22 | ns | 0.90 | ns |
|  | Wk6 | 1.53 | ns | 0.48 | ns |
|  | Wk8 | 3.36 | **** | 0.19 | ns |
|  | W12 | 5.52 | **** | 3.44 | *** |
| miR-199-3p | Wk4 | 1.19 | ns | 0.96 | ns |
|  | Wk6 | 1.56 | * | 0.52 | ns |
|  | Wk8 | 3.27 | **** | 0.14 | ns |
|  | W12 | 4.52 | **** | 2.96 | *** |
| miR-214 | Wk4 | 1.59 | ns | 0.51 | ns |
|  | Wk6 | 3.24 | ** | 1.12 | ns |
|  | Wk8 | 3.29 | ** | 0.24 | ns |
|  | Wk12 | 4.36 | **** | 8.45 | **** |
| mir-210 | Wk4 | 1.50 | ns | 0.73 | ns |
|  | Wk6 | 1.17 | ns | 0.98 | ns |
|  | Wk8 | 2.65 | ** | 1.28 | ns |
|  | Wk12 | 2.65 | *** | 5.14 | **** |
| miR-21 | Wk4 | 1.23 | ns | 1.10 | ns |
|  | Wk6 | 1.86 | **** | 0.62 | ns |
|  | Wk8 | 2.14 | **** | 1.42 | ns |
|  | Wk12 | 2.03 | **** | 2.62 | **** |
| miR-122 | Wk4 | 0.86 | ns | 0.93 | ns |
|  | Wk6 | 1.03 | ns | 0.36 | ns |
|  | Wk8 | 0.65 | ** | 0.20 | ns |
|  | Wk12 | 0.40 | **** | 0.22 | ns |
| miR-192 | Wk4 | 0.84 | * | 1.11 | ns |
|  | Wk6 | 0.98 | ns | 0.45 | ns |
|  | Wk8 | 0.50 | **** | 0.25 | ns |
|  | Wk12 | 0.37 | **** | 0.81 | ns |
| miR-194 | Wk4 | 0.88 | ns | 0.73 | ns |
|  | Wk6 | 0.70 | ns | 0.30 | ns |
|  | Wk8 | 0.51 | ** | 0.39 | ns |
|  | Wk12 | 0.42 | **** | 0.30 | ns |
| miR-365 | Wk4 | 1.04 | ns | 0.63 | ns |
|  | Wk6 | 0.79 | ns | 0.36 | ns |
|  | Wk8 | 0.63 | ns | 0.21 | ns |
|  | Wk12 | 0.82 | ns | 1.20 | ns |
