# Supplementary material for: Parasite-Derived MicroRNAs in Host Serum As Novel Biomarkers of Helminth Infection
Source: PLoS Negl Trop Dis. 2014 Feb 20;8(2):e2701. doi: 10.1371/journal.pntd.0002701 (PMC3930507; doi:10.1371/journal.pntd.0002701)
Supplement: Table S5 — Relative expression of parasite miRNAs in serum during the time course of S.mansoni infection, based on qRT-PCR analysis, normalized to values in naïve mice. (DOCX) [file pntd.0002701.s010.docx]

Table S5: Relative expression of parasite miRNAs in serum during the time course of *S.mansoni* infection, based on qRT-PCR analysis, normalized to values in naïve mice.

| miRNA |  | Background | Wk4 | Wk6 | Wk8 | Wk12 |
| --- | --- | --- | --- | --- | --- | --- |
| bantam | Mean | 1 | 7.51 | 13 | 18.55 | 27.85 |
|  | Std. Deviation | 1.23 | 13.64 | 18.13 | 22.21 | 30.28 |
|  | Holm-Sidak's multiple comparisons test |  | ns | ns | * | ** |
| miR-277 | Mean | 1 | 3.32 | 3.23 | 4.96 | 8.02 |
|  | Std. Deviation | 1.04 | 3.94 | 2.86 | 1.93 | 4.74 |
|  | Holm-Sidak's multiple comparisons test |  | ns | ns | ** | **** |
| miR-3479 | Mean | 1 | 2.69 | 3.71 | 10.02 | 22.58 |
|  | Std. Deviation | 1.02 | 1.28 | 4.73 | 11.62 | 12.42 |
|  | Holm-Sidak's multiple comparisons test |  | ns | ns | * | *** |
| miR-2a | Mean | 1 | 780.50 | 262.60 | 1755 | 3334 |
|  | Std. Deviation | 2.46 | 1412 | 497.8 | 2379 | 5061 |
|  | Holm-Sidak's multiple comparisons test |  | ns | ns | * | *** |
| miR-n1 | Mean | 1 | 0.74 | 0.87 | 10.88 | 33.29 |
|  | Std. Deviation | 1.81 | 0.62 | 1.29 | 10.13 | 27.82 |
|  | Holm-Sidak's multiple comparisons test |  | ns | ns | ns | **** |
|  | Mean | 1 | 0.01 | 2.67 | 5.62 | 8.81 |
| miR-n2 | Std. Deviation | 1.65 | 0.03 | 2.95 | 6.79 | 6.02 |
|  | Holm-Sidak's multiple comparisons test |  | ns | ns | * | *** |
|  | Mean | 1 | 11.89 | 12.02 | 14.83 | 3.76 |
| miR-n3 | Std. Deviation | 1.57 | 8.97 | 13.45 | 24.66 | 6.05 |
|  | Holm-Sidak's multiple comparisons test |  | ns | ns | ns | ns |
|  | Mean | 1 | 0.13 | 0.17 | 10.95 | 2.77 |
| miR-71 | Std. Deviation | 2.96 | 0.14 | 0.15 | 28.82 | 3.73 |
|  | Holm-Sidak's multiple comparisons test |  | ns | ns | ns | ns |
|  | Mean | 1 | 4.05 | 0.49 | 9.23 | 1.45 |
| miR-2162 | Std. Deviation | 1.52 | 8.7 | 0.43 | 22.38 | 1.40 |
|  | Holm-Sidak's multiple comparisons test |  | ns | ns | ns | ns |
